# Fermented *Astragalus membranaceus* could promote the liver and intestinal health of juvenile tiger grouper (*Epinephelus fuscoguttatus*)

**DOI:** 10.3389/fphys.2023.1264208

**Published:** 2023-09-13

**Authors:** Jingru Yang, Shengjie Zhou, Zhengyi Fu, Bo Xiao, Minghao Li, Gang Yu, Zhenhua Ma, Humin Zong

**Affiliations:** ^1^ Key Laboratory of Efficient Utilization and Processing of Marine Fishery Resources of Hainan Province, Sanya Tropical Fisheries Research Institute, Sanya, China; ^2^ South China Sea Fisheries Research Institute, Chinese Academy of Fishery Sciences, Guangzhou, China; ^3^ College of Science and Engineering, Flinders University, Adelaide, SA, Australia; ^4^ National Marine Environmental Center, Dalian, China

**Keywords:** feed additives, Chinese herbal medicine, probiotics fermentation, histology, intestinal microbiota, *Astragalus membranaceus*

## Abstract

In order to understand the effects of fermented *Astragalus membranaceus* (FAM) on the liver and intestinal health of tiger grouper (*Epinephelus fuscoguttatus*), this study was conducted. This study evaluates the effects of different levels of FAM on liver and intestinal tissue structure, serum biochemical parameters, intestinal digestive enzyme, and microbiota structure of tiger grouper. Fish were fed with diets (crude protein ≥ 48.0%, crude fat ≥ 10.0%) with five levels of FAM (L1:0.25%, L2: 0.5%, L3: 1%, L4: 2% and L5: 4%) in the experimental groups and a regular diet was used as the control (L0: 0%) for 8 weeks. Compared with AM, the protein content of FAM was significantly changed by 34.70%, indicating that a large amount of bacterial protein was produced after AM fermentation, and its nutritional value was improved. FAM had significant effects on the growth performance of tiger grouper (*p* < 0.05). The high-density lipoprotein cholesterol (HDL-C) was highest in L4 group, being significantly different from L0 group. The area and diameter of hepatocytes were lowest in L3 and L4, and the density of hepatocyte was highest in L4 group and relatively decreased in L5 group. The mucosal height and muscular thickness were highest in L3 group. The intestinal microbiota structure of tiger grouper was changed under the intervention of FAM. The lower abundance of potential pathogenic bacteria and higher abundance of probiotics colonization in the L4 group showed that the dose of FAM had the best effect on improving the health of intestinal microbiota. This study indicates that the addition of FAM in the feed contributes to liver health, improves intestinal morphology, and regulates the intestinal microbiota of tiger grouper. The addition ratio of 1%–2% is better for intestinal and liver health, and a high addition ratio will cause liver damage. Our work will provide a reference for the addition and management of FAM in the aquaculture industry.

## 1 Introduction

Tiger grouper is aquaculture-targeted specie as a result of its high commercial value and demand (Lim et al., 2016). However, the culture of tiger grouper presents many challenges due to its high susceptibility to infectious diseases and sensitivity to stress. The use of antibiotics and chemotherapeutants to control diseases of grouper as practiced by some farmers have raised several issues including high operational cost, emergence of drug-resistant bacteria, suppression of immunity, and food and environmental contamination (Apines-Amar et al., 2012). Since antibiotics and chemical drugs can promote antibiotic resistance in bacteria, they have a negative impact on the environment and human health (Meng et al., 2023), resulting in the quality of aquatic products cannot be guaranteed, which seriously affects the sustainable development of aquaculture. Although it is still widely used to alleviate diseases, some countries have restricted or banned the use of antibiotics and chemical drugs (Cabello, 2006; Meng et al., 2023). Due to the limited use of antibiotics and chemical drugs, finding alternative suitable substances to enhance the health of fish has become the focus of this study.

In order to reduce the use of antibiotics in aquaculture, alternatives such as antimicrobial peptides and probiotic feed have been proposed (Rajanbabu and Chen, 2011; Bidhan et al., 2014). In addition, Chinese herbal medicine (CHM) therapy can also enhance fish resistance, improve growth and feed efficiency, thereby enhancing the sustainability of aquaculture (Reverter et al., 2014). CHM is not only a rich source of nutrients, vitamins and minerals but also comprise several phytochemicals’ constituents which are the bioactive components like alkaloids, steroids, flavonoids, saponins, glycosides, carotenoids, terpenoids, phytoandrogens, phytosterols, curcumin and so on (Faheem et al., 2020; Mulat et al., 2020). Because of the presence of above-mentioned components, medicinal plants naturally possess several pharmacological properties making them a promising candidate as fish feed additive (Faheem et al., 2022).

Studies have shown that CHM may maintain the balance of intestinal microbiota, improve the immunity of aquatic animals, and enhance the host’s resistance to pathogen infection (Pu et al., 2017; Meng et al., 2023; Yao et al., 2023). Active components of herbs are believed to improve nutrient digestibility, absorption, assimilation capacity, and digestive enzyme secretion, as well as maintain healthy intestinal microflora in fish (Hoseinifar et al., 2020). A large number of studies have reported the beneficial effects of *Acanthopanax senticosus*, *Licorice root* and *Astragalus membranaceus* (AM) in aquaculture (Wu, 2020; Adineh et al., 2021; Meng et al., 2023). AM, a leguminous plant, is one of the most famous CHM that has various therapeutic effects such as anti-cancer, anti-virus, and immune regulation (Yao et al., 2023). Studies have shown that dietary AM supplementation can significantly improve the physiological and nutritional status of fish (Hoseinifar et al., 2020), such as catla (*Catla catla*) (Harikrishnan et al., 2022), *Channa argus* (Zhu et al., 2021), common carp (*Cyprinus carpio*) (Shi et al., 2022) and grass carp (*Ctenopharyngodon idellus*) (Shi et al., 2021).

Due to the complex structure of CHM, its active components cannot be completely absorbed and utilized by aquatic animals. The bioactivity of the active components could be seriously reduced by the traditional extraction method (Pu et al., 2017). Biological fermentation processing of traditional CHM is the use of microorganisms with strong ability to decompose and transform substances, and can produce abundant secondary metabolites. The process reaction conditions are mild, and the traditional CHM preparation is efficient, low toxicity and low residue. Therefore, the application of modern biotechnology fermentation transformation of traditional CHM has become a research hotspot (Li et al., 2004; Ruan et al., 2009). The study of Xie (2015) found that fermented CHM and CHM had obvious preventive and therapeutic effects on the hemorrhagic disease of crucian carp caused by *Aeromonas hydrophila*, and the effect of fermented CHM was better than that of unfermented CHM. Compared with liquid fermentation, solid fermentation is more and more used in clinic because of its low production cost, simple operation and no need for large instruments and equipment (Qiao et al., 2018a).

Probiotics fermentation can decompose CHM into useful components such as organic acids and polysaccharides. The fermentation products can improve the immunity, disease resistance and antioxidant capacity of the host. As an important organ of aquatic organisms, the intestine is accountable for nutrient digestion and absorption. In recent years, the intestinal microbiota has been verified that it plays a vital role in the health of the host, as it can control the proliferation of pathogenic bacteria present in the intestinal tract, regulate the host metabolism and physiology, regulate the absorption of nutrients and stimulate the immune system (Shi et al., 2022a). Hence, the intestine is not merely a vital organ responsible for the absorption of nutrients but also a major site of host immunity (Ran et al., 2020). Studies have shown that FAM can regulate the fecal microbiota of *Arbor acre*, improve its antioxidant properties and promote its growth (Qiao et al., 2018b). FAM water extract can improve the intestinal morphology and microenvironment of *Cyprinus carpio*, enhance its immune function and promote its growth (Shi et al., 2022a).

Hoseinifar et al. (2020) study found that the effects of herbal feed supplements are species-specific and must be considered cautiously. Adding 0.1% *Astragalus* compound CHM fermentation products to the feed can significantly improve the disease resistance of grass carp (*Ctenopharyngodon idella*) and significantly reduce the lethal rate of pathogenic *Aeromonas hydrophila* to it (Li and Zhou, 2018). The growth performance and survival rate of juvenile *Palea steindachneri* were significantly improved by adding 0.4% FAM to the diet, and the feed utilization rate was improved. The weight gain rate, specific growth rate and antioxidant properties of *Litopenaeus vannamei* can be significantly improved by adding 1%–4% compound probiotics FAM to the basic feed of *Litopenaeus vannamei* (Qi et al., 2018). The addition of 0.25%–2% FAM feed could significantly improve liver antioxidant performance, significantly reduce liver malondialdehyde (MDA) content and serum glutamic oxalic aminotransferase (GOT) activity of tiger grouper (Xiao et al., 2023). The addition of compound CHM fermentation preparation powder to largemouth bass (*Micropterus salmoides*) feed can improve immunity, enhance antioxidant capacity and promote fat metabolism, and the addition amount below 0.5% will not affect the production performance (Peng, 2019). The addition of 5‰–10‰ FAM feed to hybrid sturgeon can significantly improve its ability to resist streptococcal infection, and 10‰ FAM can improve its non-specific immune function (Dai, 2022). According to the application of fermented CHM in other aquatic animals and pre-experiments, we selected the inclusion levels.

Zhao et al. (2021a) and Englezos et al. (2019)' research showed that it was difficult for a single strain to complete numerous biochemical reaction processes in the fermentation process, so mixed bacterial fermentation gradually developed, and more studies were conducted on Chinese herbal compounds. *Bacillus subtilis* can produce many enzymes such as cellulase, protease and hemicellulase during its growth (Liu et al., 2012). In the early stage of fermentation, *Bacillus subtilis* grows and multiplies rapidly and consumes oxygen. With the reduction of oxygen, *Lactobacillus plantarum* begins to grow and multiply, produce lactic acid, and gradually reduce the pH value, and effectively control the infection of other miscellaneous bacteria (Jung et al., 2012; Li et al., 2021a). The growth conditions of *Saccharomyces cerevisiae* are extensive and easy to cultivate. It can use the fermentation products of *Bacillus subtilis*, such as hexose and pentose (Xie, 2015), to produce protein and multiple vitamins, and remove the product effect. *Lactobacillus plantarum* and *Enterococcus faecalis* have a good symbiotic relationship (Qiao et al., 2018a). The former provides the latter with essential peptides and amino acids, such as glycine, histidine, valine, leucine, glutamic acid, tryptophan and isoleucine (Bassaganya-Riera et al., 2012), while the latter produces formic acid and CO_2_ to stimulate the proliferation of the former. Among many species of *Aspergillus fungi*, *Aspergillus flavus* can produce toxic substances and cannot be used in microbial transformation. *Aspergillus Niger* has a variety of highly active enzyme systems, does not produce toxins, can produce pectinase, mannanase, protease, amylase, cellulase, hemicellulase, lipase, glucosidase and other enzymes, it is widely used in the conversion of traditional Chinese medicine (Jin et al., 2016). In addition to *Aspergillus Niger*, other Aspergillus enzymes such as *Aspergillus oryzae* have also been applied, but they are far less than the application of *Aspergillus Niger*. So in this experiment, *Astragalus* was fermented by mixed bacteria (*Aspergillus Niger spores*, *Bacillus subtilis*, *Saccharomyces cerevisiae*, *Lactobacillus plantarum* and *Entero-coccus faecalis*) solid-state fermentation process. In aquaculture, the use of probiotics and dietary enhancement have been recognized as alternative methods of health management. In particular, nutritional status has been increasingly acknowledged as a crucial factor in host defense against pathogens. As such, use of feed supplements aiming to improve not only the growth but also the health of aquaculture species has gained widespread interest and acceptance. Our work will provide reference for the addition and management of FAM in the aquaculture industry.

## 2 Materials and methods

### 2.1 Fermented *Astragalus membranaceus* (FAM) and diet preparation

#### 2.1.1 Fermentation process

Chinese herbal medicine AM produced in Gansu and fermented by mixed bacteria (*Aspergillus Niger spores*, *Bacillus subtilis*, *Saccharomyces cerevisiae*, *Lactobacillus plantarum* and *Entero-coccus faecalis*) solid-state fermentation process. The fermentation method was improved according to Xie (2015)’s method and previous studies (Liu et al., 2017; Qiao et al., 2018a; Li et al., 2021a). AM, corn, soybean meal and wheat bran were pulverized and passed through a 100mesh sieve, and then mixed at 75: 10: 10: 5 as the initial material. In addition, 5% molasses, 0.2% ammonium sulfate, 0.05% potassium dihydrogen phosphate, 0.1% dipotassium hydrogen phosphate, 0.07% sodium chloride and 0.01% magnesium sulfate heptahydrate were added. Drying at 55°C for 24 h before mixing. The substrate was sterilized by 121°C high temperature and high-pressure steam (SHENAN, LDZX-30KBS, China) for 20min and then added into the sterile fresh water of equal quality. *Aspergillus Niger* spores’ powder (2 × 10^10^ CFU·g^−1^), *Bacillus subtilis* powder (2 × 10^11^ CFU·g^−1^), *Saccharomyces cerevisiae* powder (2 × 10^10^ CFU·g^−1^), *Lactobacillus plantarum* powder (1 × 10^10^ CFU·g^−1^) and *Enterococcus faecalis* powder (1 × 10^11^ CFU·g^−1^) were used for fermentation, the inoculum concentration of each strain was 2 × 10^7^ CFU·g^-1^. FAM preparation conditions were as follows: aerobic fermentation at 35°C for 24°h, anaerobic fermentation at 35°C for 72°h, drying at 55°C for 24°h, and grinding to particle size < 0.15°mm.

#### 2.1.2 Feed preparation

The basic feed is commercial grouper feed, produced in Santong Bio-engineering (Weifang) CO., Ltd. (http://www.santonghaitong.com/). The nutritional levels of feed were as follows: crude protein ≥ 48.0%, crude fat ≥ 10.0%, crude ash ≤ 16.0%, crude fiber ≤ 3.0%, lysine ≥ 2.5%, moisture ≤ 10.0%. Commercial complete crushed to particle size < 0.28mm, 1.5% adhesive sodium alginate was added. FAM or microcrystalline cellulose (the total addition was 4%) were added to make granules, drying at 55°C for 24 h. The diet was stored at −20°C.

The control group (L0) was fed commercial complete feed with no added FAM. Five treatments were involved. The treatment group (L1, L2, L3, L4 and L5) was fed commercial complete feed supplemented with FAM (0.25%, 0.5%, 1%, 2% and 4%, respectively). For each group, three replicates were used. During the experimentation period, 30% water was exchanged in each tank. The water parameters were measured daily and were maintained at pH 7.8, dissolved oxygen > 6.0 mg/L, and water temperature 26.5°C ± 1.5°C.

### 2.2 Chemical characterization analysis

*Astragalus* dry powder and FAM were weighed three equal samples to be tested. Protein content was determined by Kjeldahl nitrogen determination method (GB 5009.5-2016). Polysaccharide was extracted by water extraction and alcohol precipitation method (Qiao, 2020), total sugar was determined by phenol-sulfuric acid method (Zhao, 2015), and reducing sugar was determined by 3, 5-dinitrosalicylic acid colorimetric method (Zhang et al., 2017). Polysaccharide extraction rate (%) = (total sugar mass - reducing sugar mass)/sample mass×100% (Chen, 2020). The relative molecular mass of polysaccharide was determined by gel permeation chromatography (GPC) method (Yang et al., 2023).

The content of total saponins was determined by sulfuric acid-vanillin method and total flavones by sodium nitrite—aluminum nitrate-sodium hydroxide method (Zhang et al., 2021). The content of Astragaloside A was determined by HPLC-ELSD method according to Pharmacopoeia of the People’s Republic of China (2020 edition). The content of effective substances in FAM containing the same amount of AM = the content of effective substances in FAM ÷ 70.85%.

### 2.3 Experimental fish and feeding management

Juvenile tiger grouper individuals (bodyweight 44.48 ± 2.06 g) were raised at the Tropical Fisheries Research and Development Center, South China Sea Fisheries Research Institute, Chinese Academy of Fishery Sciences, Sanya, China. We randomly divided 540 fish among 18 tanks (300 L each, 30 fish per tank). Fish were allowed to acclimate for 7 days and were only fed commercial complete feed during this period. Upon completion of the acclimation period, experimentation began and lasted 8 weeks. Fish were fed *ad libitum* twice daily at 08:30 and 16:30.

### 2.4 Fish performance and feed efficiency

At the end of the feeding trial, following a 24 h starvation period and anesthetized with 150 mg/L Eugenol CAS:97-53-0, all the fish were measured and weighed. In addition, 3 fish from each tank were randomly collected before sampling. The calculation formulas of fish performance and feed efficiency are as follows:

$$\text{Weight gain rate }\left(\text{WGR},\%\right)=\left(W_{t}-W_{0}\right)\;/W_{0}\times 100$$

$$\text{Specific growth rate }\left(\text{SGR},\%\cdot d^{-1}\right)=\left({\text{lnW}}_{t}-{\text{lnW}}_{0}\right)\;/t\times 100$$

$$\text{Survival rate }\left(\text{SR},\%\right)=N_{f}/N_{i}\times 100$$

$$\text{Feed coefficient }\left(\text{FC}\right)=F/\left(W_{t}-W_{0}\right)$$

In the formula, W_0_ was the fish body weight (g) at the beginning of the experiment; W_t_ was the fish weight (g) at the end of the experiment; t was the test days (d); N_f_ was the number of terminal fish; N_i_ was the initial fishtail number; F was feed intake (g).

### 2.5 Sampling

The surface water was wiped with paper towels, and a 1-mL sterile syringe was used to extract blood from the tail veins of tiger grouper. After standing for 4 h, blood samples were centrifuged for 10 min at 3500×g, 4°C, and then the supernatants were collected. The collected supernatant was stored at −80°C for further measurement and analysis. The intestines and livers of three fish from each tank were aseptically dissected; the intestinal contents were collected.

### 2.6 Physiology and biochemistry

Serum biochemical parameters were determined according to the instructions of the commercial kits (Nanjing Jiancheng Biological Co., Ltd., Nanjing, China), i.e., total protein (TP) (Item No. A045-4-2): BCA microplate method; total cholesterol (TC) (Item No. A111-1-1): cholesterol oxidase–peroxidase aminoantipyrine method; high density lipoprotein cholesterol (HDL-C) (Item No. F003-1-1): liquid precipitation separation method; and low-density lipoprotein cholesterol (LDL-C) (Item No. A113-1-1): double reagent direct method. All serum biochemical parameters were performed in triplicates.Intestine samples from each parallel were weighed. Under the condition of ice bath, 0.9% normal saline or sample homogenate medium was added to the tissue according to the weight volume ratio of 1:9 to make 10% homogenate. According to the requirements of the corresponding kits (Nanjing Jiancheng Biological Co., Ltd., Nanjing, China), the homogenates were centrifuged and the supernatants were extracted, and then the relevant indicators were measured. Intestinal digestion indicators included amylase (AMS) (Item No. C016-1-1), lipase (LPS) (Item No. A054-2-1), trypsin (TRYP) (Item No. A080-2-2), and total protein (TP) (Item No. A045-4-2). The starch-iodine colorimetric method, methyl halal substrate method (microplate method), colorimetric method, and BCA microplate method were used for determination, respectively.

### 2.7 DNA extraction

The intestinal contents from the three fish were mixed, transferred to a sterile freezing tube, snap-frozen in liquid nitrogen, and stored at −80°C (Haier, DW-86L626, China) for DNA extraction. Total DNA was extracted from the intestinal contents using TIANamp Stool DNA Kits (Tiangen), following the manufacturer’s instructions. A ultramicro biochemical spectrophotometer (Thermo Scientific, NanoDrop, 2000, China) and agarose gel electrophoresis (Beijing Liuyi Instrument Factory, DYY-6C, China) were used to determine DNA quantity and quality.

### 2.8 PCR amplification and 16S rRNA gene library construction

The V3–V4 hypervariable region of the bacterial 16S rRNA gene was PCR-amplified using universal primers (338F: 5′-ACT​CCT​ACG​GGA​GGC​AGC​AG-3′, 806R: 5′-GGACTACHVGGGTWTCTAAT-3′). Indexed adapters were added to the ends of the 16S rRNA gene amplicons to generate indexed libraries for downstream NGS sequencing on the Illumina MiSeq platform. Sequencing adapters were also added to the termini of the PCR products to facilitate MiSeq sequencing. All PCR amplifications were performed in triplicate using TransStart FastPfu DNA Polymerase Kits (TransGen). Each 20ul PCR mixture contained 4ul of 5×FastPfu Buffer, 2.5 ul of dNTPs, 0.8 ul of each primer, 0.4 ul of FastPfu Polymerase, 0.2 ul of BSA, 10 ng of template DNA, and ddH2O to make 20ul. The thermal cycling conditions were as follows: initial denaturation at 95°C for 3min; 27 cycles of denaturation at 95°C for 30 s, annealing at 55°C for 30 s, extension at 72°C for 45 s; and a final extension at 72°C for 10 min. All of the PCR products were visualized on agarose gels (2% in TAE buffer) containing ethidium bromide and purified using DNA gel extraction kits (Axygen).

### 2.9 Bioinformatics analysis

After de-multiplexing the data and discarding certain reads, the remaining reads were converted to FASTQ format. In this study, 250-bp reads were truncated at any site receiving an average quality score of < 20 over a 10bp sliding window. Reads < 50bp were discarded. The minimum value of the overlap was 10bp when merging the reads; sequences whose barcodes did not match an expected barcode were also discarded.

Chimeric sequences were determined by UCHIME (Edgar et al., 2011). OTUs were defined with a threshold of 97% similarity by UPARSE (Edgar, 2013). Taxonomic richness and diversity estimators were determined for each library in Mothur. The mean of the estimated richness was used for comparisons among samples. The heatmap was constructed by using the heatmap 2 function of the R g-plots package based on the top 100 genera of the samples.

### 2.10 Histological analysis

The livers and intestines were collected and fixed in 4% paraformaldehyde. The fixed tissues were embedded in paraffin blocks and sliced into a series of transverse sections (4μm thick) using a Leica RM 2016 rotary microtome (Shanghai Leica Instrument Co., Ltd., China). A hematoxylin–eosin (HE) stain was used for general histological analysis. Each slide with tissue sections was mounted permanently using neutral balsam. The sections were scanned using a Panoramic 250/MIDI scanner (3D HISTECH Co., Ltd., Hungary), and Case viewer 2.0 (3D HISTECH Co., Ltd., Hungary) was used for image analysis and measurement. For each intestine sample, mucosal height and muscularis thickness were quantified by taking 10 measurements per intestinal section. For each liver sample, area, density and diameter of hepatocyte were quantified by taking 10 measurements per liver section.

### 2.11 Statistical analysis

Statistical analysis was performed using SPSS 19.0 statistical software package (SPSS Inc.). Excel 2016 for data processing and mapping. Microbioinformatics analysis and mapping were performed on the Meiji Biocloud platform (https://cloud.majorbio.com/) of Shanghai Meiji Biotechnology Co., Ltd. All of the values are presented as means ± standard deviation (mean ± SD). *t*-test was used to analyze the significant difference of *Astragalus* components before and after fermentation. One-way analyses of variance (ANOVAs) were used to analyze the data of growth performance, morphological index, serum biochemical index, digestive enzyme and intestinal flora α diversity test results. Comparisons between different groups were conducted by LSD test when there was a significant difference. We considered *p* < 0.05 statistically significant.

## 3 Results

### 3.1 Chemical characterization

#### 3.1.1 Components

As shown in Table 1, compared with *Astragalus* herb, the total saponin content, polysaccharide extraction rate, total flavonoid content, astragaloside A and protein content of FAM were significantly different (*p* < 0.05), which were differed by 87.44%, 21.77%, 200%, 34.07% and 34.70%, respectively. After mass conversion, the total saponin content, polysaccharide extraction rate, astragaloside A content and total flavonoid content in FAM containing the same amount of AM were significantly different (*p* < 0.05), which were differed by 164.73%, 71.88%, 6.94% and 340%, respectively.

**TABLE 1:** Composition changes of *Astragalus* membranaceus before and after fermentation.

Items	*Astragalus* herb	FAM	Effective substance content after mass conversion
Total saponin content/%	2.07 ± 0.05	3.88 ± 0.06*	5.48 ± 0.09*
Polysaccharide extraction rate/%	9.28 ± 0.06	11.30 ± 0.37*	15.95 ± 0.53*
Total flavone content/%	0.05 ± 0.00	0.15 ± 0.00*	0.22 ± 0.01*
Astragaloside A content/mg·kg^−1^	1726.26 ± 25.39	1138.13 ± 22.08*	1606.39 ± 31.16*
Protein content/%	13.63 ± 0.12	18.36 ± 0.67*	—

Note: “*” indicates significant difference compared with *Astragalus* herb (*p* < 0.05, *n* = 9). FAM: fermented *Astragalus*.

#### 3.1.2 Relative molecular mass of polysaccharides

**FIGURE 1:**
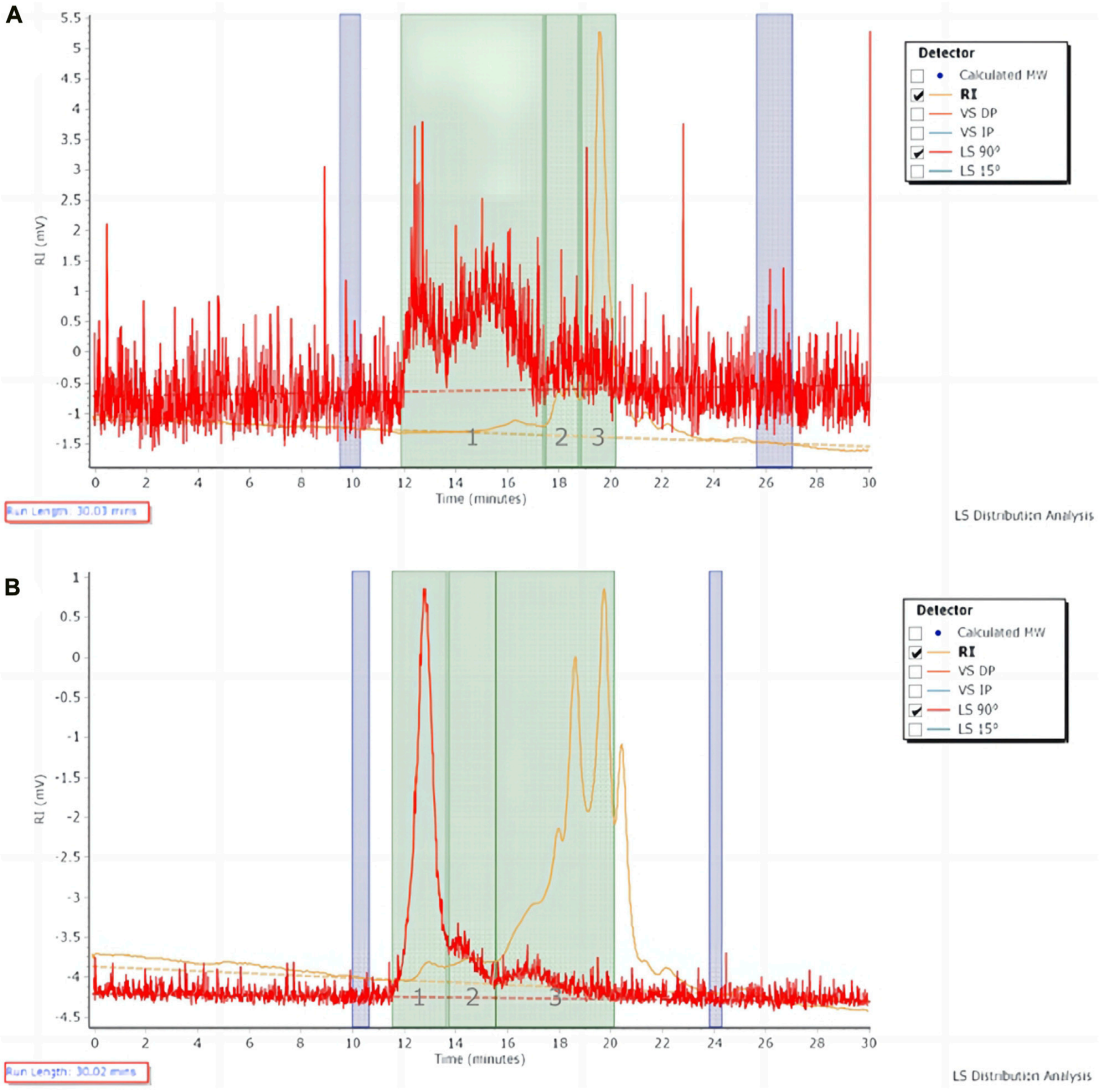
The chromatogram of polysaccharide in *Astragalus* herb and fermented *Astragalus*. **(A)** *Astragalus* herb; **(B)** Fermented *Astragalus*.

The molecular weight of the extracted polysaccharide of *Astragalus* herb and FAM was determined by GPC method. The polysaccharide with larger molecular weight entered the micropores of the filler less and was separated out first. The longer the retention time, the smaller the average molecular weight of the polysaccharide (Figure 1). The peak molecular weight (Mp), weight-average molecular weight (Mw), number-average molecular weight (Mn) and molecular weight (MW) distribution of polysaccharide samples on the chromatogram were calculated by GPC software through the viscosity and peak time of the samples (Tables 2, 3). The peak dispersion coefficient (Mw/Mn) data before and after fermentation showed that the molecular weight distribution of *Astragalus* polysaccharides before and after fermentation was uneven, and the distribution range was wide, including three different molecular weight components. However, the molecular weight distribution of *Astragalus* polysaccharides before and after fermentation was significantly different (*p* < 0.05). The highest molecular weight of the component represented by peak 1 after fermentation was 117634167 larger than that before fermentation, and the proportion of molecular weight above 1000000Da was 51.15%, which was also higher than that before fermentation, indicating that *Astragalus* promoted the dissolution of large molecular weight polysaccharides after fermentation. According to the difference in the relative proportion of the peak area of peak 3 to the total peak area before and after fermentation, it can be seen that the relative peak area of *Astragalus* polysaccharide peak 3 changed significantly after fermentation (*p* < 0.05), indicating that the fermentation of *Astragalus* promoted the degradation and utilization of large molecular weight polysaccharides by probiotics, which was converted into small molecular weight polysaccharides, resulting in an increase in the proportion of small molecular weight polysaccharides. The proportion of components represented by peak 3 with a molecular weight below 10000Da after fermentation also showed the same results.

**TABLE 2:** Molecular weight determination result of polysaccharide in *Astragalus* herb and fermented *Astragalus*.

Samples	Peak number	Mp/Da	Mw/Da	Mn/Da	Mw/Mn
Polysaccharide of *Astragalus* herb	1	214819	364948	144580	2.524194
	2	54556	57901	57490	1.007149
	3	3578	39672	29834	1.329758
Polysaccharide of FAM	1	1669612	1679129	662840	2.533234
	2	59647	68658	52987	1.295752
	3	6511	11602	9467	1.22552

Note: FAM, fermented *Astragalus*; Mp, peak molecular weight; Mw, weight-average molecular weight; Mn, number-average molecular weight.

**TABLE 3:** Molecular weight distribution of *Astragalus* herb and fermented *Astragalus*.

Peak number		MW		MW		Percent/%	
		High limit MW/Da		Low limit MW/Da			
*Astragalus* herb	FAM	*Astragalus* herb	FAM	*Astragalus* herb	FAM	*Astragalus* herb	FAM
1	1	96741174	117634167	1000000	1000000	7.03	51.15
1	1	1000000	1000000	500000	500000	11.36	20.54
1	1	500000	500000	300000	300000	13.74	13.86
1	1	300000	300000	200000	200000	16.55	10.81
1	1	200000	200000	100000	175512	26.14	3.63
1		100000	—	50000	—	21.12	—
1		50000	—	43241	—	4.06	—
2	2	70060	155622	50000	100000	99.47	19.97
2	2	50000	100000	49909	50000	0.53	40.63
	2	—	50000	—	30000	—	28.01
	2	—	30000	—	24453	—	11.39
3	3	151226	36971	100000	30000	3.54	2.08
3	3	100000	30000	50000	20000	16.84	8.92
3	3	50000	20000	30000	10000	39.27	37.35
3	3	30000	10000	20000	5431	23.83	51.65
3		20000	—	10000	—	16.47	—
3		10000	—	9971	—	0.04	—

Note: FAM, fermented *Astragalus*; MW, molecular weight.

### 3.2 Growth performance

Feed addition of FAM had no significant effect on the survival rate of tiger grouper. The WGR and SGR were highest in L4 group, being significantly different from all other treatments, including L0 group (*p* < 0.05). However, when the dose of FAM was the highest (L5 group), the WGR and SGR again decreased. The FC was lowest in L3 and L4 group, being significantly different from all other treatments, including L0 group (*p* < 0.05). However, when the dose of FAM was the highest (L5 group), the FC again increased (Table 4).

**TABLE 4:** Effects of fermented *Astragalus* on growth performance of tiger grouper.

Items	L0	L1	L2	L3	L4	L5
SR/%	98.89 ± 1.92	98.89 ± 1.92	98.89 ± 1.92	100 ± 0.00	100 ± 0.00	100 ± 0.00
WGR/%	75.09 ± 6.38^c^	84.27 ± 5.65^bc^	77.76 ± 8.16^bc^	90.18 ± 9.53^b^	105.00 ± 7.10^a^	79.42 ± 5.13^bc^
SGR/%·d^−1^	1.00 ± 0.06^c^	1.09 ± 0.06^bc^	1.03 ± 0.08^bc^	1.15 ± 0.10^b^	1.28 ± 0.06^a^	1.04 ± 0.05^bc^
FC	1.31 ± 0.17^a^	1.23 ± 0.18^a^	1.40 ± 0.18^a^	0.93 ± 0.03^b^	0.93 ± 0.07^b^	1.24 ± 0.04^a^

Note: The control group (L0) was fed commercial complete feed with no added FAM, The treatment group (L1, L2, L3, L4 and L5) was fed commercial complete feed supplemented with FAM (0.25%, 0.5%, 1%, 2% and 4%, respectively). SR, survival rate; WGR, weight gain rate; SGR, specific growth rate; FC, feed coefficient. In the same row, values with different small letter superscripts mean significant difference (*p* < 0.05, *n* = 9).

### 3.3 Serum biochemical parameters

The HDL-C was highest in L4 group, being significantly different from all other treatments, including L0 group (*p* < 0.05). Feed addition of FAM had no significant effect on TP, TC and LDL-C of tiger grouper (Table 5).

**TABLE 5:** Effects of fermented *Astragalus* on serum biochemical indexes of tiger grouper.

Items	L0	L1	L2	L3	L4	L5
TP/g·L^−1^	11.55 ± 1.05	11.87 ± 0.73	12.25 ± 0.67	12.15 ± 0.44	12.33 ± 1.20	11.08 ± 0.75
TC/mmol·L^−1^	3.27 ± 0.25	3.23 ± 0.14	3.19 ± 0.13	3.18 ± 0.11	3.04 ± 0.26	3.19 ± 0.33
HDL-C/mmol·L^−1^	4.61 ± 0.16^b^	5.06 ± 0.51^b^	4.89 ± 0.72^b^	5.02 ± 0.73^b^	5.53 ± 0.42^a^	5.22 ± 0.20^b^
LDL-C/mmol·L^−1^	1.80 ± 0.24	1.72 ± 0.49	1.66 ± 0.17	1.70 ± 0.17	1.59 ± 0.15	1.62 ± 0.07

Note: The control group (L0) was fed commercial complete feed with no added FAM, The treatment group (L1, L2, L3, L4 and L5) was fed commercial complete feed supplemented with FAM (0.25%, 0.5%, 1%, 2% and 4%, respectively). TP, total protein; TC, total cholesterol; HDL-C, high density lipoprotein cholesterol; LDL-C, low-density lipoprotein cholesterol. In the same row, values with different small letter superscripts mean significant difference (*p* < 0.05, *n* = 9).

### 3.4 Digestive enzymes

**FIGURE 2:**
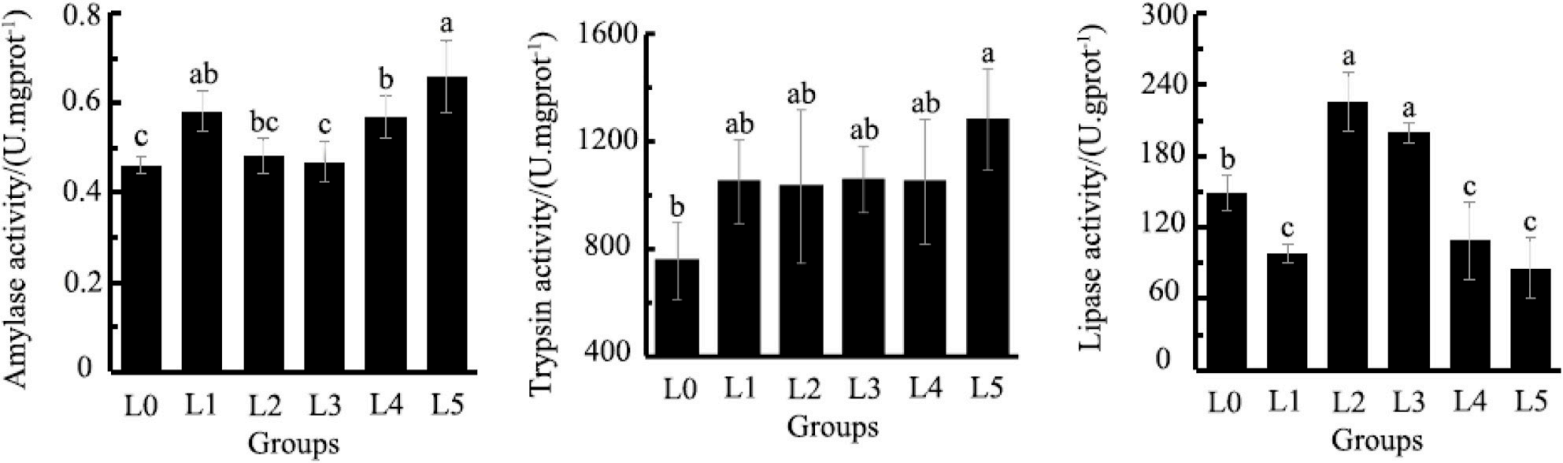
Effects of Fermented *Astragalus* on intestinal amylase activity, trypsin activity and lipase activity of tiger grouper. Note: The control group (L0) was fed commercial complete feed with no added FAM. The treatment group (L1, L2, L3, L4 and L5) was fed commercial complete feed supplemented with FAM (0.25%, 0.5%, 1%, 2% and 4%, respectively). Different superscript letters indicate significant differences among treatments (*p* < 0.05).

The AMS was lowest in L0, being significantly different from L1, L4 and L5 (*p* < 0.05). The TRYP was lowest in L0, being significantly different from L5 (*p* < 0.05). The LPS was highest in L2 and L3, being significantly different from all other treatments, including L0 group (*p* < 0.05) (Figure 2).

### 3.5 Hepatic histology

The area and diameter of hepatocyte were lowest in L3 and L4, being significantly different from L1 (*p* < 0.05). The density of hepatocyte was highest in L4 group, being significantly different from all other treatments, including L0 group. However, when the dose of FAM was the highest (L5 group), the density of hepatocyte again decreased (Table 6).

**TABLE 6:** Effects of fermented *Astragalus* on liver morphological parameters of tiger grouper.

Items	L0	L1	L2	L3	L4	L5
Area of hepatocyte/um^2^	63.23±	79.51±	72.00±	48.16±	47.98±	68.97±
	13.08^ab^	7.14^a^	1.69^ab^	8.74^b^	11.57^b^	31.07^ab^
Density of hepatocyte/(Number/mm^2^)	3249.39±	3582.26±	3989.97±	3849.22±	4590.92±	3653.75±
	182.63^d^	66.01^c^	26.88^b^	209.82^bc^	220.97^a^	275.12^bc^
Diameter of hepatocyte/um	8.75±	9.71±	9.12±	7.95±	7.75±	8.87±
	0.75^ab^	0.37^a^	0.10^ab^	0.54^b^	0.67^b^	1.48^ab^

Note: The control group (L0) was fed commercial complete feed with no added FAM, The treatment group (L1, L2, L3, L4 and L5) was fed commercial complete feed supplemented with FAM (0.25%, 0.5%, 1%, 2% and 4%, respectively). In the same row, values with different small letter superscripts mean significant difference (*p* < 0.05, *n* = 9).

**FIGURE 3:**
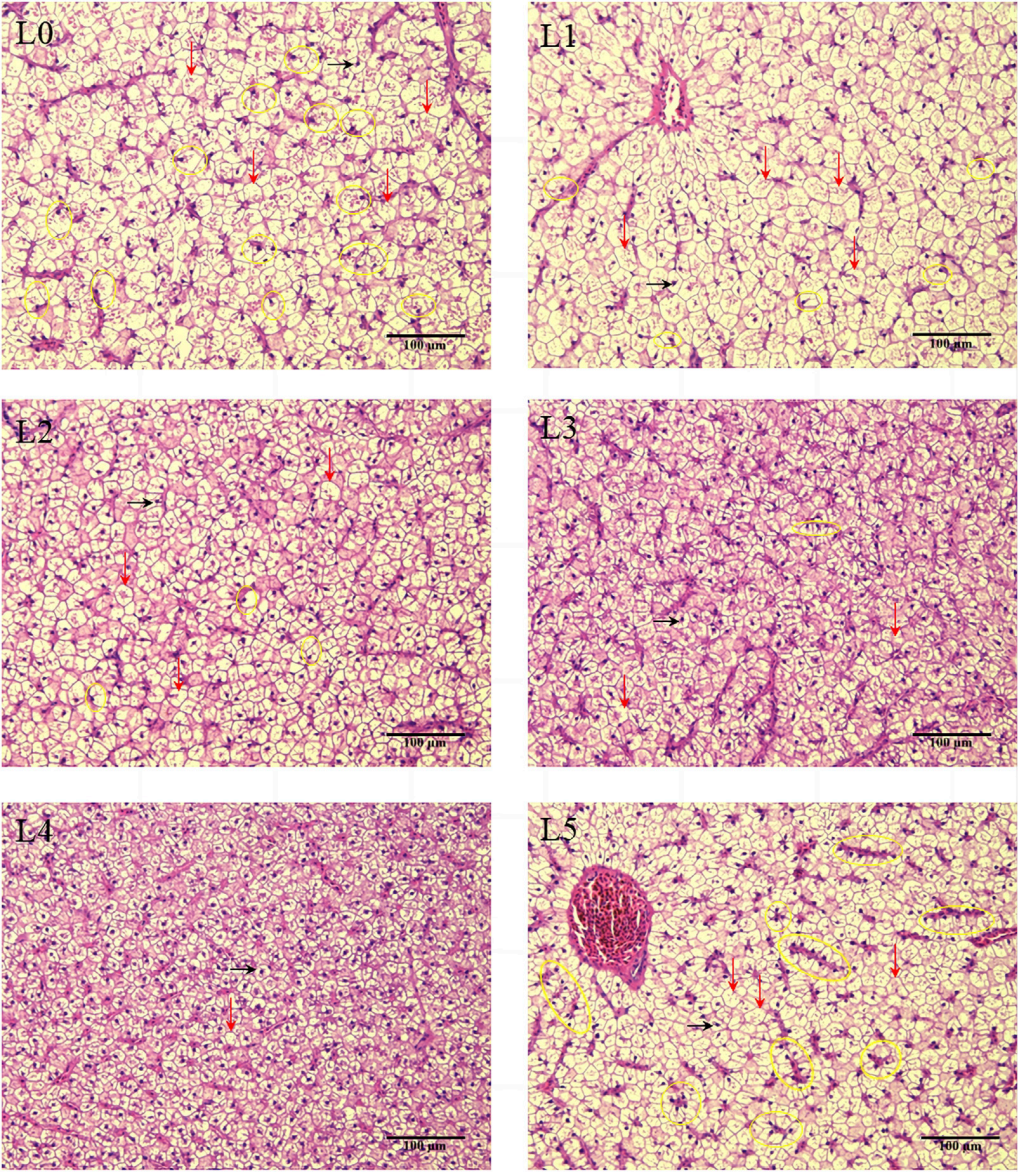
Effects of fermented *Astragalus* on hepatic tissue structure of tiger grouper. Note: The control group (L0) was fed commercial complete feed with no added FAM. The treatment group (L1, L2, L3, L4 and L5) was fed commercial complete feed supplemented with FAM (0.25%, 0.5%, 1%, 2% and 4%, respectively). The black arrow marks the liver nucleus, the black ellipse marks the shift of the liver nucleus, and the red arrow marks the hepatocyte swelling and vacuolation.

The results of HE staining of liver tissue sections of tiger grouper was shown in Figure 3. In the L0 group, many hepatocytes were swollen and vacuolated, some nuclei were deviated, and the number of nuclei in the field of view was less than other groups (from density of hepatocyte). After the addition of FAM, the migration of hepatocyte nucleus in L1 group was reduced, but the cell swelling and vacuolization were not improved. In the L2 group, the number of hepatocyte nuclei increased significantly, a small number of nuclei shifted, and cell swelling and vacuolization were improved. Cell swelling, vacuolization and nuclear migration in the L3 group were further improved. In the L4 group, only a very few hepatocytes showed vacuolization and nuclear deviation, cell swelling was significantly improved, clear nuclei were visible in the center of the cells, and the number in the field of vision was significantly increased. The hepatocytes were evenly distributed and closely arranged, and the cell membrane contour was clear. The L5 group with a high proportion of FAM showed swelling and vacuolization, and there was a serious nuclear deviation. The number of nuclear deviation cells was significantly higher than that of other groups, showing a pathological feature.

### 3.6 Intestinal histology

The measurement results of tiger grouper intestinal tissue sections in each group were shown in Table 7. The mucosal height was highest in L3 group, being significantly different from L0, L1, L2 and L4 groups (*p* < 0.05). The muscularis thickness was highest in L3 and L5 group, being significantly different from L0, L1 and L2 groups (*p* < 0.05). Feed addition of FAM had no significant effect on the mucosal number of tiger grouper.

**TABLE 7:** Effects of fermented *Astragalus* on intestinal morphological parameters of tiger grouper.

Items	L0		L1	L2	L3	L4	L5
Mucosal height	355.60 ± 135.23b		346.64 ± 170.09^b^	314.85 ± 144.72^b^	447.79 ± 252.91^a^	363.55 ± 149.26^b^	402.38 ± 157.20^ab^
Muscularis thickness	131.24 ± 27.12ab		113.93 ± 22.89^b^	115.20 ± 34.51^b^	154.85 ± 63.44^a^	134.05 ± 43.47^ab^	143.79 ± 29.01^a^
Mucosal number	32.33 ± 3.06		30.33 ± 1.53	30.00 ± 2.65	34.00 ± 6.08	33.33 ± 8.50	31.33 ± 0.58

Note: The control group (L0) was fed commercial complete feed with no added FAM, The treatment group (L1, L2, L3, L4 and L5) was fed commercial complete feed supplemented with FAM (0.25%, 0.5%, 1%, 2% and 4%, respectively). Different superscript letters indicate significant differences among treatments (*p* < 0.05, *n* = 9).

**FIGURE 4:**
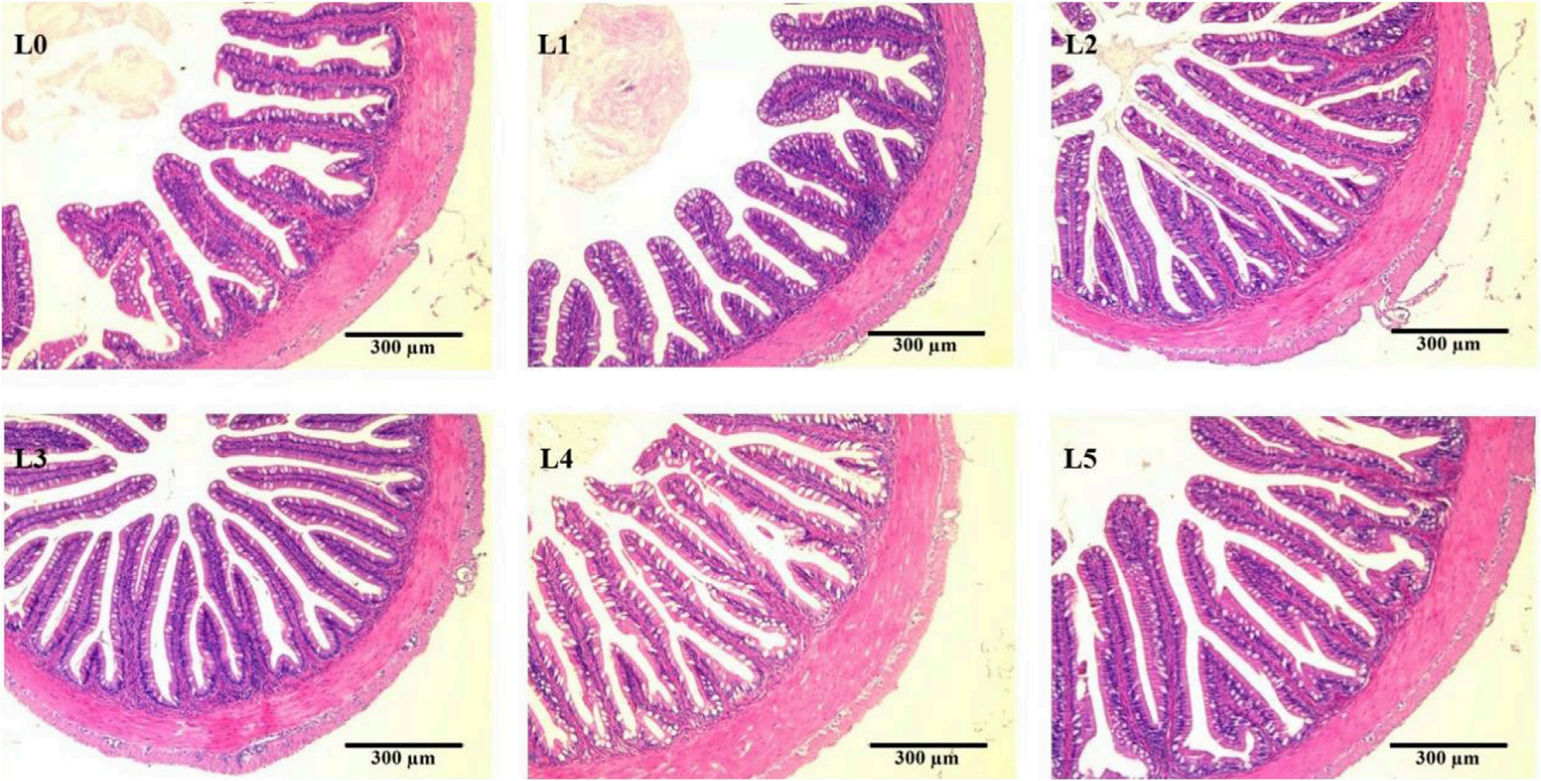
Effects of fermented *Astragalus* on intestinal morphology of tiger grouper. Note: The control group (L0) was fed commercial complete feed with no added FAM. The treatment group (L1, L2, L3, L4 and L5) was fed commercial complete feed supplemented with FAM (0.25%, 0.5%, 1%, 2% and 4%, respectively). h, mucosal height; t, muscularis thickness; black circle indicates the mucosa; red circle indicates the dissolution and shedding of columnar epithelial cells.

At the same time, FAM affected the intestinal microstructure of tiger grouper, as shown in Figure 4. In the control group, the intestinal mucosa was sparse and disordered, and the columnar epithelial cells of the intestinal mucosa were dissolved and separated from the lamina propria. The nucleus at the base was disordered, and the intestinal mucosa microstructure was significantly damaged. Compared with the control group, the damage to the intestinal structure in each FAM group was alleviated, and the effect of the L3 group was better. The intestinal tissue structure was complete and clear. The intestinal mucosa was well developed, arranged closely and abundantly, and straight into the cavity. The nuclei of epithelial cells were closely and neatly arranged at the base, and the striated edges were neatly arranged.

### 3.7 Intestinal microbiota structure

#### 3.7.1 Intestinal microbial OTU division and Alpha diversity comparison

**FIGURE 5:**
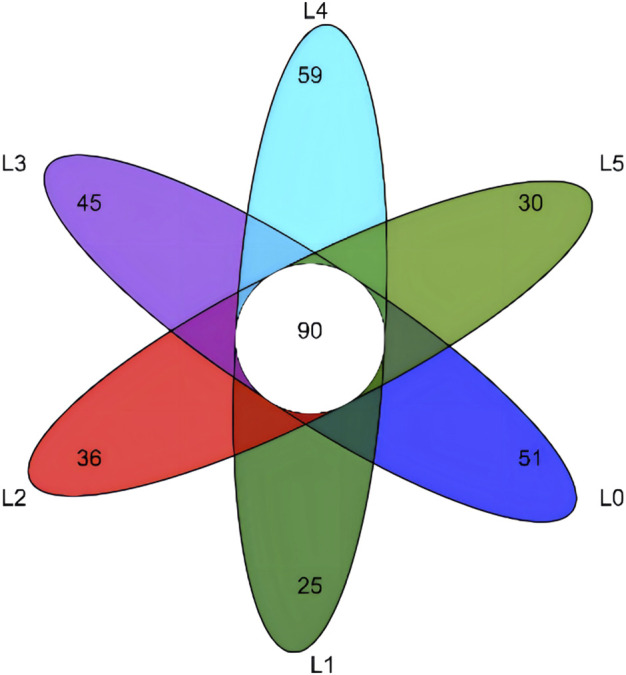
Venn diagram of intestinal microbiota of tiger grouper. Note: The control group (L0) was fed commercial complete feed with no added FAM. The treatment group (L1, L2, L3, L4 and L5) was fed commercial complete feed supplemented with FAM (0.25%, 0.5%, 1%, 2% and 4%, respectively).

The sequences with similarity higher than 97% were clustered into the same classification operation unit (OTU) for bioinformatics statistical analysis. From the OUT-classification level, draw the Venn diagram, as shown in Figure 5. A total of 336 OTUs were obtained from the test samples, of which the number of common OTUs was 90. The only OTUs in the L0-L5 group were 51, 25, 36, 45, 59 and 30, respectively. The total OTU values of each group were 141, 115, 126, 135, 149 and 120, respectively. Therefore, the ratio of the only OTU to the total OTU was 36.17%, 21.74%, 28.57%, 33.33%, 39.60% and 25.00%, respectively. The above results can be concluded that the structure of tiger grouper intestinal microbiota can be changed due to the intervention of FAM. The richness and diversity of intestinal microbial communities were analyzed by single sample diversity (Alpha diversity), including a series of statistical analysis indexes to estimate the species abundance and diversity of ecological communities. The Alpha diversity analysis of this experiment was estimated by five indexes. The results are shown in Table 8. At the level of 97% similarity, the sequencing coverage of each sample was higher than 99.8%. There was no significant difference in the Sob index, Shanno index, Simpson index, Ace index and Chao index between groups (*p* > 0.05).

**TABLE 8:** Alpha-diversity indexes of gut microbiota of tiger grouper.

Groups	Sob index	Shanno index	Simpson index	Ace index	Chao index
L0	148.33 ± 69.30	2.11 ± 0.76	0.28 ± 0.11	165.25 ± 65.31	175.61 ± 66.65
L1	133.00 ± 13.53	2.25 ± 0.31	0.23 ± 0.14	146.10 ± 8.74	147.57 ± 12.22
L2	139.33 ± 13.58	1.98 ± 0.26	0.30 ± 0.07	143.18 ± 12.98	144.21 ± 11.87
L3	136.00 ± 10.15	1.76 ± 0.64	0.43 ± 0.18	141.60 ± 6.64	146.89 ± 6.60
L4	170.33 ± 3.21	2.53 ± 0.37	0.29 ± 0.09	176.37 ± 3.35	179.44 ± 7.85
L5	141.33 ± 28.57	1.87 ± 0.56	0.36 ± 0.13	146.18 ± 28.42	147.46 ± 30.23

Note: The control group (L0) was fed commercial complete feed with no added FAM, The treatment group (L1, L2, L3, L4 and L5) was fed commercial complete feed supplemented with FAM (0.25%, 0.5%, 1%, 2% and 4%, respectively).

#### 3.7.2 Intestinal microbial community composition and difference analysis

**FIGURE 6:**
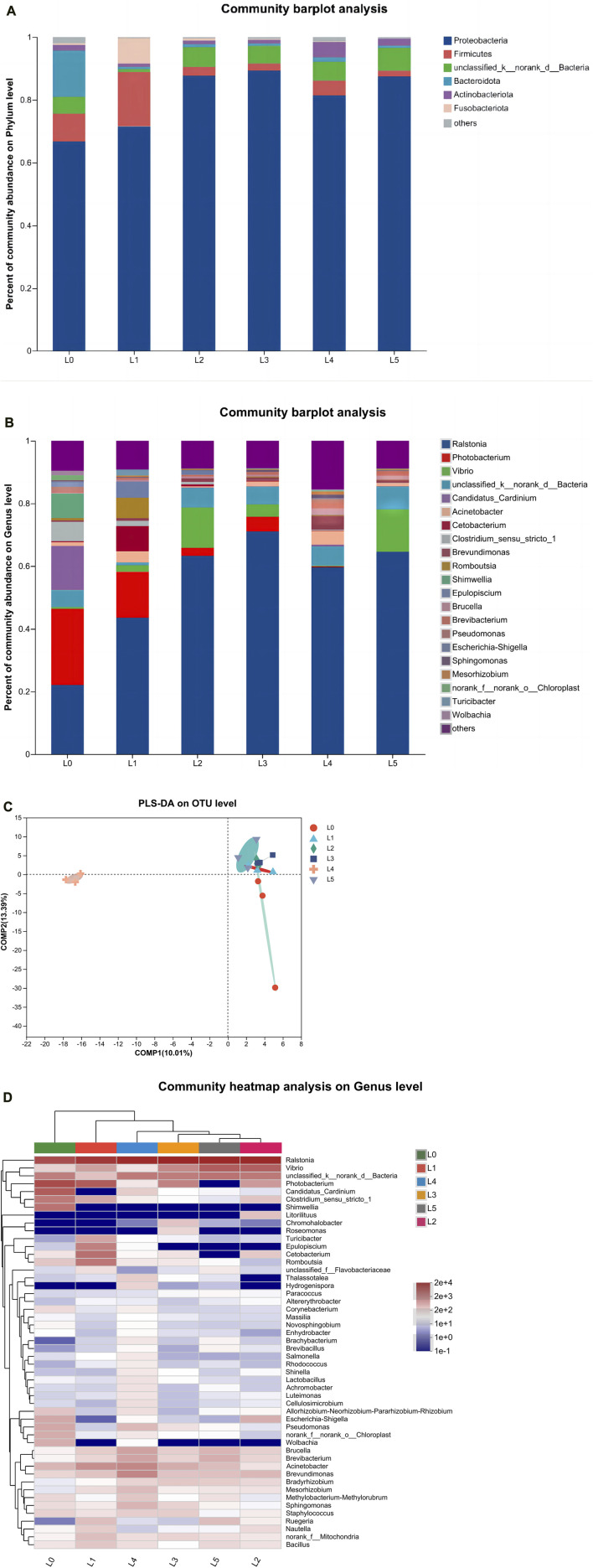
Intestinal microbiota of tiger grouper. **(A)** Community composition at phylum level; **(B)** Community composition at genus level; **(C)** PLS-DA **(D)** The clustering heat map analysis at the genus level. Note: The control group (L0) was fed commercial complete feed with no added FAM. The treatment group (L1, L2, L3, L4 and L5) was fed commercial complete feed supplemented with FAM (0.25%, 0.5%, 1%, 2% and 4%, respectively).

As shown in Figure 6A, *Proteobacteria*, *Firmicutes*, Unclassified _ k __ norank _ d __ Bacteria, *Bacteroidota*, *Actinobacteria* and *Fusobacteriota* were the dominant phyla in tiger grouper at the phylum level. *Proteobacteria* (66.78%), *Firmicutes* (8.87%) and *Bacteroidota* (14.71%) were dominant in the L0 group. *Proteobacteria* (71.56%), *Firmicutes* (17.36%) and *Fusobacteriota* (8.15%) were dominant in the L1 group. The composition of dominant bacteria in L2-L4 groups was similar. The proportion of *Proteobacteria* in each group was 87.70%, 89.33%, 81.44% and 87.41%, respectively. The proportion of *Firmicutes* in each group was 2.81%, 2.28%, 4.77% and 1.91%, respectively. The proportion of Unclassified _ k __ norank _ d __ Bacteria in each group was 6.27%, 5.60%, 6.00% and 7.30%, respectively. In addition, the proportion of *Actinobacteria* in the L4 group was significantly higher than that in other groups, reaching 4.81%. The above shows that *Proteobacteria* was the first dominant phylum.

The relative abundance of tiger grouper intestine microbiota at the genus level is shown in Figure 6B. At the genus classification level, there were some differences in the dominant intestinal microbiota of each treatment group. The relative abundance of core microbiota was higher in *Ralstonia*, *Photobacterium*, *Vibrio*, Unclassified _ k __ norank _ d __ Bacteria and *Candidatus_Cardinium*, which constituted the dominant genus in the intestinal microbiota of tiger grouper in this experiment. The abundance of *Ralstonia* and *Vibrio* were lowest in L0 group, being significantly different from all other treatments, except L4 group.

According to Figure 6C Partial Least Squares Discriminant Analysis (PLS-DA), the control group was significantly different from the experimental group in terms of species abundance, while the L4 group was more significantly different from the other groups, indicating that FAM had a specific effect on the intestinal microbiota of tiger grouper, and the L4 group had the most significant effect.

As shown in Figure 6D, according to the abundance information and species annotation of the samples at the level of intestinal bacterial genus, the genus with abundance in the top 50 was screened. According to the abundance information in each sample, the samples and species were clustered at two levels to draw a heat map. The color gradient in the heat map analysis from blue to red indicates that the relative abundance is from low to high. The results showed that the intestinal microbial samples of each experimental group were first clustered into one branch, which was far away from the L0 group. The abundance of *Ralstonia* in the control group was lowest. In addition to the L4 group, the *Vibrio* abundance of each experimental group was also higher than that of the L0 group. The abundance of *Photobacterium*, *Pseudomonas*, *Wolbachia*, *Escherichia-Shigella* and *Shimwellia* in each experimental group was lower than that in the control group. The abundance of *Rhodococcus*, *Shinella*, *Lactobacillus*, *Achromobacter*, *Brucella*, *Brevibacterium*, *Acinetobacter* and *Brevundimonas* in the L4 group was higher than that in other groups. Except for the L3 group, the abundance of *Bacillus* in each experimental group was relatively higher than that in the L0 group.

## 4 Discussion

Modern pharmacological studies have shown that AM has anti-cancer, analgesic, anti-inflammatory, antibacterial, antioxidant and other pharmacological effects, and contains a variety of active ingredients such as saponins, flavonoids, polysaccharides, amino acids and trace elements. AM is one of the most widely used traditional herbs. The main functions of *Astragalus* polysaccharides and flavonoids are anti-oxidation and immunity enhancement. Saponins have the functions of anti-tumor, immune regulation, and protection of the cardiovascular system. Astragaloside A is the main active ingredient of *Astragalus* saponins, which is a qualitative and quantitative index stipulated in the pharmacopoeia. In this experiment, AM was fermented by mixed bacteria solid-state fermentation process. After fermentation, it had a special fermented koji aroma. Compared with the crude drug AM, the protein content of FAM was significantly changed by 34.70%, indicating that a large amount of bacterial protein was produced after AM fermentation, and its nutritional value was improved. The total saponin content, polysaccharide extraction rate and total flavonoid content in FAM containing the same amount of AM were significantly changed by 164.73%, 71.88% and 340%, but the content of astragaloside A was significantly changed by 6.94%. Similar probiotic fermentation experiments of AM showed that the use of *Aspergillus Niger*, *Lactobacillus plantarum*, and *Saccharomyces cerevisiae* for mixed solid-state fermentation of AM, the polysaccharide extraction rate and total flavonoid content of AM increased by 11.93% and 43%, respectively (Zhou, 2018). The liquid fermentation of AM roots was carried out by *non-lactose streptococcus*, and the increase rates of polysaccharides, total saponins and total flavonoids were 177.46%, 68.50% and 55.67%, respectively (Su, 2017). Wang (2020) used liquid fermentation of AM by *Bacillus natto*. After fermentation, the polysaccharide content decreased by 28.49% compared with that before fermentation, the total saponin increased by about 9.21%, and the total flavonoid content increased by about 36.43% (Wang, 2020); Zhao (2015) used *lactic acid bacteria* to ferment AM by solid-state fermentation. The content of polysaccharide and astragaloside A were the highest on the 12th and 18th days, respectively, and the increase rates were 95.5% and 17.46% higher than those before fermentation, but lower than the control group in the later stage of fermentation (Zhao, 2015); Qin (2012) used the selected lactic acid bacteria to ferment AM in liquid state, and the yield of polysaccharide increased by 59.34%, but the extraction rate of total saponins decreased by 17.20% after fermentation, and the content of astragaloside A decreased by 27.6% (Qin, 2012). The above results showed that different fermentation methods had different effects on the yield of effective components of AM. Under the action of powerful enzymes of microorganisms, dense structural components such as cellulose, hemicellulose and pectin in plant cell wall are decomposed and transformed, resulting in loose structure of plant cell wall, increased intercellular space and easy release of active components, so the content of active components is increased (Song et al., 2021). However, some chemical components may also be bio transformed by probiotics, thus changing the chemical composition of traditional Chinese medicine (Ai et al., 2019). In this study, the content of astragaloside A in FAM decreased, indicating that the fermented strain may have been bio transformed with this precursor. The specific transformation pathway and the new compounds generated need to be further studied. As one of the extremely important active ingredients of *Astragalus*, *Astragalus* polysaccharides have pharmacological effects such as anti-virus, regulating blood sugar, anti-oxidation and enhancing immunity (Wang et al., 2022b). The use of gel chromatography to detect the molecular weight changes of *Astragalus* polysaccharides before and after fermentation helps to evaluate its efficacy changes. In this experiment, the yield of polysaccharides increased after fermentation, and the proportion of polysaccharides in the low molecular weight section changed significantly after fermentation, indicating that the high molecular weight polysaccharides were degraded after fermentation, which was consistent with the results of Liang Zijing FAM polysaccharides (Liang, 2019). High molecular weight polysaccharides have poor water solubility, complex structure and conformation, and are difficult to cross the tissue barrier into the cell or attach to the receptor to play a role (Zhang, 2020). On the contrary, low molecular weight polysaccharides have higher solubility and lower viscosity than high molecular weight polysaccharides in water, so they are more easily absorbed by the body when they act in the body, have higher bioavailability, and have higher affinity with phagocytes, which contributes to immune activation (Li et al., 2020); In addition, polysaccharides with lower molecular weight also have higher antioxidant activity (Sun et al., 2009). The proportion of low molecular weight polysaccharides was significantly increased after fermentation of AM. The results provide a reference for the functional characteristics of FAM, such as immune regulation and intestinal microecology improvement.Enzymes produced in the process of microbial fermentation can effectively decompose plant cell wall, so that the effective components can be released from the cell to improve the medicinal effect of traditional Chinese herbal medicine (Hussain et al., 2016). A large number of studies had shown that fermented Chinese medicine can improve the growth performance of aquatic animals, such as common carp (*Cyprinus carpio*) (Zhao et al., 2017; Shi et al., 2022a), *Cyprinus carpio haematopterus* (Xie et al., 2015) and Ctenopharyngodon idella (Tang et al., 2021). During the experiment, the WGR and SGR were highest in L4 group, the FC was lowest in L3 and L4 group, being significantly different from all other treatments, including L0 group. The comprehensive performance of the L3 and L4 group was better, indicating that FAM can promote the digestion and absorption of nutrients in fish and promote the growth of tiger grouper, which was similar to the results of *Palea steindachneri* (Nong and Li, 2019) and common carp (*Cyprinus carpio*) (Shi et al., 2022a). The polysaccharide yield of Astragalus was improved after probiotic fermentation (Xiao et al., 2023). As one of the effective active ingredients of *Astragalus*, *Astragalus* polysaccharide can promote the growth of fish has been reported (Xiang et al., 2011; Wang et al., 2018). In addition, the probiotics added during the fermentation process can secrete a variety of digestive enzymes after activation, growth and reproduction, thereby improving intestinal health and improving the digestion and absorption capacity of animals (Xiao et al., 2023). The nutritional factors such as bacterial protein, vitamins, and small peptides produced during the metabolic process may also have a positive effect on the growth of the test fish. Studies had shown that adding 1.5% *Astragalus* directly to *Pseudosciaena crocea* R. feed causes more than half of the mortality rate (Wei, 2014). In this experiment, the addition of FAM had no significant effect on the survival rate of tiger grouper, and the highest dose of the L5 group (4% FAM) also had no death, indicating that the fermentation treatment of AM may reduce the toxic and side effects of high-dose traditional Chinese medicine on fish. The reason is that the microbial population in fermentation decomposes and transforms the anti-nutritional components of traditional Chinese medicine or modifies the structure of toxic substances, thus improving the pharmacological characteristics of Chinese herbal medicine (Li et al., 2021b), which was also one of the advantages of fermented traditional Chinese medicine. In general, the addition of FAM at 1%–2% had a better effect on promoting growth and reducing feed ratio.

The metabolism and physiological and pathological conditions of fish can be reflected by serum biochemical indicators (Zhou et al., 2001). Liver is the main place for protein synthesis, and serum TP concentration can reflect the ability of liver protein synthesis and metabolism (He et al., 2017). Serum TC is mainly synthesized in the liver, reflecting the liver’s metabolism of fat (Tan et al., 2018). Cholesterol transport depends on high-density lipoprotein and low-density lipoprotein. High-density lipoprotein helps transport cholesterol from peripheral tissues and plasma to the liver, degrades and removes excess cholesterol to maintain cholesterol homeostasis in the body (Meng et al., 2021). Low-density lipoproteins transport endogenous cholesterol synthesized in the liver to peripheral tissues for their use (Wang et al., 2021a). Feed addition of FAM had no significant effect on TP, TC and LDL-C of tiger grouper. The HDL-C was highest in L4 group, being significantly different from all other treatments, including L0 group. The above results indicate that FAM can promote the lipid transport in the liver of tiger grouper.

The liver is the main metabolic organ of fish and plays an important role in regulating physiological functions, such as digestion, nutrient storage, synthesis of new substances, detoxification of harmful chemicals and metabolic homeostasis (He, 2019; Zhong et al., 2021). In order to pursue the benefits of aquaculture, high-energy long-term feeding can lead to liver dysfunction in fish, which in turn induces fatty liver and metabolic disorders, eventually leading to slow growth of fish (Tan et al., 2019) Previous studies have shown that plants or plant extracts can increase liver lipid metabolism and improve liver morphology in fish (Tan, 2020; Sun et al., 2022). Compared with the control diet, *Pangasianodon hypophthalmus* juveniles fed with AM extract significantly reduced liver injury index enzymes and improved liver health (Abdel-Latif et al., 2022). Similar results were obtained in juvenile crucian carp (*Carassius auratus*) fed with AM polysaccharide (Wu, 2020). The protective effect of *Astragalus* polysaccharides on common carp (*Cyprinus carpio*) liver cells was verified in the model of liver injury induced by carbon tetrachloride (CCl_4_) (Jia et al., 2012). And the FAM polysaccharides also had antagonistic effects on liver injury and liver fibrosis in rats (Qin, 2012). In the present research, studies have shown that *Astragalus* active substances can prevent the negative effects of internal or external factors on the liver, and then play its liver protection role. In this study, histological examination showed that hepatocytes in group L0 fed only artificial basal diet showed swelling vacuolization and nuclear shift, indicating that the liver integrity of tiger grouper was sensitive to artificial basal diet. Studies have shown that compared with feeding ice fresh feed, feeding artificial basic feed can induce liver lipid accumulation, and liver histological analysis shows more vacuolization, which in turn causes liver inflammation and oxidative stress, and ultimately leads to liver injury (Ma et al., 2020), which is consistent with our research results. In the gradient addition ratio of FAM set in this experiment, the liver histology was gradually improved with the increase of dose, and the liver integrity of the L4 group was the best (cell swelling was significantly improved, and the number of cells in the nucleus was significantly increased). In the L5 group with the highest addition ratio, cell swelling and vacuolization occurred, and severe nuclear deviation occurred. The pathological characteristics showed that liver cell damage occurred in the L5 dose group. The above research results showed that adding appropriate proportion of FAM to the feed can effectively regulate the homeostasis of fat metabolism and is beneficial to liver health. However, high-dose addition may have toxic effects on the liver, destroy the structure and function of the liver, and make the liver lipid metabolism function abnormal, resulting in a certain degree of disorder in the fat transport system, but cell swelling and vacuolization. The possible reasons why FAM is beneficial to liver health are as follows. Astragalus has effective antioxidant components, such as astragaloside, flavonoids and polysaccharides, which can effectively prevent tissue damage through its antioxidant mechanism (Muhammad et al., 2016). In addition, probiotics in FAM may also be beneficial to fish liver health. The application of multiple varieties of probiotics changed the shape of liver nuclei from irregular to regular and reduced the space between liver tissues. Adding *Bacillus subtilis* solid-state fermentation products to zebrafish feed can improve liver lipid metabolism and alleviate lipid deposition (Wang et al., 2022a). The possible mechanism of probiotics improving liver health is to regulate glucose and lipid metabolism, reduce fat accumulation in the liver, regulate intestinal microbiota homeostasis, repair intestinal barrier and relieve inflammation (Yao et al., 2021).

The biological process of fermentation leads to changes in the nutritional composition of the fermentation substrate (Zhang et al., 2012). Among them, the added probiotics have many beneficial effects, such as killing or inhibiting pathogens, affecting the intestinal microflora, and promoting nutrient utilization (Gao et al., 2022), thus affecting the digestion ability of animals. The results of this experiment showed that FAM had different degrees of improvement on the activity of digestive enzymes in tiger grouper. The principle of Chinese herbal medicine as a natural growth promoter may be to induce the secretion of digestive enzymes and stimulate appetite (Zhang et al., 2015), and to cooperate with the probiotics added by fermentation to improve the intestinal microenvironment. The addition of FAM in this experiment increased the activity of digestive enzymes in tiger grouper and reduced the feed coefficient, indicating that the reason why the addition of FAM in the feed accelerated the growth rate of tiger grouper was not by increasing its food intake, but by enhancing its digestion and absorption capacity.The intestinal morphology of fish is closely related to dietary components (Wang et al., 2017; Fu et al., 2021; Yang et al., 2022). The complete intestinal structure is crucial for the body to prevent pathogens and toxins from entering the systemic circulation, absorb and utilize nutrients, and resist environmental pressure (Yang et al., 2022). It is an important guarantee for the growth rate and health status of aquatic animals. The intestinal mucosa formed by the epithelial cells of the intestinal mucosa is the basic structure of the intestinal tract. The height, quantity, morphological structure and sparse degree of the intestinal mucosa and the development degree of the striated edge on the surface have a great influence on the homeostasis of the intestinal environment and the digestion and absorption of nutrients (Khosravi et al., 2015). Intestinal epithelial cells, tight junction proteins and mucus layer constitute the intestinal barrier function (Yan and Ajuwon, 2017). In this experiment, compared with the control group, the addition of FAM reduced the dissolution and shedding of columnar epithelial cells of intestinal mucosa, improved the morphology of intestinal tissue, significantly increased the height and number of intestinal mucosa and the thickness of muscular layer, the addition ratio of 1% was the best. The reason may be that short-chain fatty acids (Qiao, 2020) or other prebiotics are produced during the fermentation process of AM, which promotes the growth of beneficial bacteria, inhibits harmful bacteria, improves the homeostasis of intestinal environment, and thus facilitates the development of intestinal mucosa (Cheng et al., 2021). The improvement of intestinal morphology enhances the intestinal barrier function, improves the immune and pathogenic microbial defense ability, and may reduce intestinal cell apoptosis (Wu et al., 2022a), and ultimately has a positive impact on the body’s nutrient utilization and growth (Yang et al., 2022).

The intestinal microbiota of fish is essential for maintaining the health of aquatic animals (Wang et al., 2021b). Intestinal microbiota is a key factor affecting various functions of the host, including development, digestion, growth, disease resistance and immunity (Burr et al., 2005). Therefore, it is very important to explore how to change the intestinal microbiota when feeding tiger grouper FAM. The results of this experiment showed that the microbial composition of the intestinal tract of tiger grouper changed significantly with the 8-week breeding experiment. Except that the diversity and richness of the L4 group showed an upward trend compared with the other groups, FAM had no significant effect on the Alpha diversity index of intestinal microbiota, but each group in this experiment had a considerable proportion of unique OUT, indicating that FAM changed the composition of intestinal microbial community. At the phylum level, *Proteobacteria* was the absolute dominant phylum in each group, the relative abundance in the control group was 66.78%, and the relative abundance in each treatment group increased by more than 70%. *Bacteroidota* was significantly enriched in the L0 group, *Fusobacteriota* was significantly enriched in the L1 group, and the *Firmicutes* enrichment abundance in the L2-L4 group was lower than that in the L0 and L1 groups. *Proteobacteria*, *Bacteroidota*, *Fusobacteriota* and *Firmicutes* represent more than 80% of intestinal microbes in various marine and freshwater species (He et al., 2018; Li et al., 2022). The similarity of this bacterial taxa indicates that intestinal microbes are involved in important host intestinal functions such as digestion of nutrients and immunity (Xu et al., 2023). The increase of *Proteobacteria* abundance may be beneficial to the absorption and utilization of feed nutrients by fish under the catabolism of feed components by bacteria (Roeselers et al., 2011; Wu et al., 2022b). *Ralstonia* (Bai et al., 2019) and *Vibrio* (Clements et al., 2014) are the main intestinal microbiota of marine fish, but *Vibrio* is also a potential pathogen of marine fish. *Ralstonia* is a potential pathogen for humans and animals (Monica et al., 2019), and no pathogenic cases have been reported in aquatic animals. Some strains of *Photobacterium*, *Pseudomonas*, *Acinetobacter*, and *Escherichia-Shigella* are often considered as potential pathogens (Behera et al., 2018; Shao et al., 2020; Li et al., 2022), and a certain number of these microbiotas exist in fish for a long time, which may cause disease when fish immunity decreases and microbiota imbalance occurs. *Bacillus* can secrete digestive enzymes and has a good inhibitory effect on pathogenic bacteria (Lu, 2021). *Lactobacillus* can metabolize to produce lactic acid and antibacterial bacteriocins, and also has an inhibitory effect on pathogens (Kaktcham et al., 2017). In addition, *Lactobacillus* can also induce the proliferation of short-chain fatty acid-producing strains in intestinal microbiota, increase the content of short-chain fatty acids in intestinal tract, and benefit intestinal health (Liu, 2019). The change trend of *Ralstonia* abundance was consistent with the growth performance. The increase of *Ralstonia* abundance may promote the growth and feed utilization of fish. The possible reason is that the synthesis of short-chain fatty acids in intestinal microbiota is enhanced after intake of FAM, and its activation enhances the energy supply of the genus bacteria. The abundance of *Photobacterium*, *Pseudomonas* and *Escherichia-Shigella* in L0 group was highest. The abundance of *Vibrio* in L4 group was lowest while the abundance of *Acinetobacter* and *Lactobacillus* in L4 group was the highest. The abundance of *Bacillus* in L3 group was lowest. The above changes in the abundance of potential pathogenic bacteria and intestinal colonization probiotics indicated that FAM regulated the complexity of intestinal microbiota structure, which may be related to its dose effect. The L4 group had relatively lower abundance of potential pathogenic bacteria and higher abundance of colonization probiotics, indicating that this dose had the best effect on improving intestinal microbiota. Healthy intestinal microbiota can improve the body‘s immunity and promote the barrier function of the intestine. It can not only secrete digestive enzymes to promote the conversion and absorption of feed nutrients, but also secrete vitamins to make up for the deficiency of feed nutrients (Wang et al., 2012), which is consistent with the results of FAM improving intestinal morphology and growth performance. Xiao et al. (2023) studies have shown that FAM has a degradation effect on Astragalus polysaccharides. Low molecular weight polysaccharides have high solubility and higher bioavailability, while potential probiotics can ferment polysaccharides or oligosaccharides to produce short-chain fatty acids (Georgina, 2014), thereby promoting fish health. Flavonoids in FAM may inhibit pathogenic bacteria and promote the growth of probiotics in the intestine, thereby regulating the structure of intestinal microbiota (Zhao et al., 2021b). In addition, the probiotics and their metabolites in FAM also affect the composition and structure of fish intestinal microorganisms, and cooperate with the physiologically active substances in Astragalus to maintain the stability and balance of intestinal microbiota.

## 5 Conclusion

The fermented *Astragalus* could promote the growth of tiger grouper and improve the feed conversion rate. FAM also could improve intestinal and liver morphology and regulate intestinal microbiota. This study shows that FAM can promote the growth performance and liver and intestinal health of tiger grouper. However, the high addition ratio of FAM may will adversely impact the fish body. Therefore, it is recommended that the addition ratio of FAM in the tiger grouper feed be 1%–2% of the diet weight.

## Data Availability

The datasets presented in this study can be found in online repositories. The names of the repository/repositories and accession number(s) can be found below: NCBI SRA under PRJNA1001328.
